# Examining the Role of Fear of Falling on Gait Parameters and Short-Term Gait Adaptation in Older Adults

**DOI:** 10.3390/jcm14238311

**Published:** 2025-11-22

**Authors:** Anna Brachman, Victoria Hadyk, Anna Kamieniarz-Olczak, Agnieszka Nawrat-Szołtysik

**Affiliations:** 1 Institute of Sport Sciences, Academy of Physical Education, Department of Biomechanics, 40-065 Katowice, Poland; 2 Institute of Sport Sciences, Academy of Physical Education, Department of Human Motor Behavior, 40-065 Katowice, Poland; v.hadyk@awf.katowice.pl (V.H.); a.kamieniarz@awf.katowice.pl (A.K.-O.); 3 Institute of Physiotherapy and Health Sciences, Academy of Physical Education, 40-065 Katowice, Poland; a.nawrat-szoltysik@awf.katowice.pl

**Keywords:** fear of falling, walking adaptability, older adults, treadmill

## Abstract

**Background**: Motor adaptation is a process where movements need to be modified in response to changes in the task, environment, or organism itself. Even a very short-term process of adaptation to different movement execution conditions leads to improvements in task performance. There is no information in the literature on whether such a gain would be present in older adults with different fear of falling levels (FoF). **Methods**: Thirty older adults with various FoF underwent short-term adaptive gait training on a treadmill. Participants’ spatiotemporal and foot loading characteristics were assessed directly before and after stimulation in two walking conditions: with preferred speed, and in challenging conditions (maximum speed). **Results**: Correlation analysis revealed moderate-to-strong associations with fear (0.66–0.74) in a majority of spatiotemporal parameters, as well as in rear and forefoot loading (0.48–0.82). In more challenging conditions, observed associations were more pronounced; however, comparative analysis revealed that the correlation was significantly stronger only in stride length (*p* = 0.03), walking speed (*p* = 0.03), forefoot (*p* = 0.01), and rearfoot loading (*p* = 0.02). There was a marginal effect of adaptive training during walking with preferred speed, but in demanding conditions, results showed significant improvements in each fear level group. **Conclusions**: The correlation between fear and plantar loading during walking in older adults implies a more cautious foot roll-over pattern with increasing FoF. Results also suggest that the relationship between fear and detrimental gait changes scales to some extent with motor task difficulty. The data suggest preserved but possibly attenuated gait adaptability in highly fearful older adults.

## 1. Introduction

Walking is a critical motor ability in daily living. With aging, multiple detrimental changes in the neuromuscular system are observed, which lead to a reduced ability to produce a safe and effective gait pattern [1,2,3]. It has been reported that changes in the sensorimotor system in healthy older adults lead to an increased involvement of executive function, like attention, information updating, or monitoring during movement production, even in actions as well-learned and automated as walking [4,5]. It has been postulated that age-associated brain changes (particularly white matter atrophy in the frontal lobes and gray tissue loss in the prefrontal cortex) lead to a decline in the aforementioned executive functions, and this decline has been associated with gait impairments [4,6,7].

The deterioration of gait and balance in older adults can be even more pronounced by psychological factors, such as fear of falling (FoF), resulting in higher fall risk, which may result in fractures, deterioration of physical function, and loss of autonomy [8,9]. FoF is defined as a concern about falling during activities of daily living, which an individual remains capable of performing, and, as a consequence, often leads to activity restriction [1,10]. FoF is a common condition among the elderly, and can progress to a stage where walking becomes cautious and needs more attentional resources [8]. Disturbances in walking in older adults with FoF manifest through the deterioration of spatio-temporal gait characteristics like shorter step, stride length, increased double support phase, wider base of support, and decreased gait speed [1,2,8]. The available literature provides limited information regarding the patterns of foot loading in individuals experiencing fear of falling. To date, only one study [11] observed an increased pressure–time integral in the total foot and decreased center of pressure velocity during stance phase progression with increased fear. Information about the force distribution and loading aspects of the feet during walking in fearful older adults is lacking. Also, the question arises as to whether these detrimental gait changes scale with task difficulty. Namely, whether the strength of associations remains at a constant level or becomes more pronounced when older individuals are placed in more challenging conditions. Answers to these questions would shed light on our understanding of the influence of fear on motor system functions and gait characteristics in older adults.

Numerous studies indicate that the capacity for motor learning and motor adaptive improvements in older adults is largely preserved [12]. Durand-Ruel et al. [13] explored behavioral and brain correlates of fast motor learning. The authors found that learning of grip force modulation in two 12 min sequential tasks yielded changes in sensorimotor network activity and improvement in accuracy and time needed to complete trials in both training sessions. Berghuis et al. [14] tested the influence of 13 min visuomotor tracking task training in young and older adults. Results showed that, although older adults’ motor performance was worse than that of young adults, the magnitude of improvement was similar over time. Correspondingly, in another study [15], 5 min training of a complex sequence of finger tapping resulted in similar performance improvement in both young and older adults, in the trained hand.

It is well known that fear influences motor performance. Researchers have suggested that anxiety can cause the reinvestment phenomenon, where regression to more novice forms of movement and more conscious movement execution of well-learned skills may occur [16,17,18]. However, little is known about how fear influences motor learning or the motor adaptation process. Lin et al. [19] investigated motor responses after exposure to a moving sled in patients with functional gait disorders and healthy controls. Patients showed a greater state of anxiety than controls, but similar normal adaptive learning. The authors also noticed a longer de-adaptation process, indicating a tendency to sustain the acquired motor pattern in patients. In a previous study [20] with a similar experimental methodology, the authors reported that fear-based mechanisms may promote locomotor learning in healthy young adults. Although these studies increase our insight into the relation between fear and motor adaptation mechanisms, none of them evaluated associations between the different fear of falling levels and the gait adaptation process in older adults. A better understanding of this relationship seems to be essential for our understanding of how the motor system functions in older adults and how to possibly improve rehabilitation processes.

Therefore, this study aims to do the following: (i) to examine the strength of associations between FOF, plantar loading, and spatiotemporal gait characteristics; (ii) to determine whether the strength of associations become more apparent under more demanding conditions, such as walking at maximum speed; (iii) to explore whether the process of learning, i.e., short-term adaptation, is affected by different fear levels.

## 2. Materials and Methods

Participants were recruited using community-based outreach methods, including collaboration with senior centers. Individuals with a high fear of falling were partially recruited from institutional settings. The data were gathered between September 2024 and March 2025. Thirty older adults (6 males, 24 females, mean age 68.8 ± 3.9 years, mass 74.5 ± 10.9 kg, height 163.4 ± 7.9 cm) met the following inclusion criteria: (1) age 60 years and older, (2) the ability to perform the measurement and training, (3) no experience in virtual reality gaming. The exclusion criteria were as follows: (1) chronic systemic and inflammatory diseases (i.e., rheumatoid arthritis or osteoarthritis); (2) neurological, cardiovascular, or orthopedic diseases affecting postural stability (i.e., severe low back pain, epilepsy, uncontrolled hypertension, vestibular disorders); (3) cognitive impairment (MMSE < 27); or (4) non-corrected visual or hearing deficits. Participants were divided into three fear level groups [21] for low (*n* = 8), moderate (*n* = 14), and high (*n* = 8) concern about falling (Table 1). The study was carried out in accordance with the guidelines proposed in the Declaration of Helsinki, and it was approved by the Institutional Ethics Committee (no. 1-VI/2024). All enrolled participants provided informed consent. The participants were also informed that they could leave the study at any time without any explanation.

### 2.1. Measurements

The short Falls Efficacy Scale International (FES-I) was used to assess the level of fear of falling (FoF). The scale has been shown to have good validity and reliability. Participants were divided into three groups: low (7–8 pts), moderate (9–13 pts), and high (14–28 pts) fear of falling [21]. Following anthropometric and FoF assessment, participants were instructed to walk on the instrumented treadmill FDM-t 3i (Zebris Medical GmbH, Isny im Allgäu, Germany). Participants were given about a 5 min treadmill acclimatization session, in which they were briefed regarding the safety procedures. During the acclimatization session, their preferred comfortable speed was determined.

During each measurement trial, participants walked barefoot for 30 s at their preferred walking speed. Immediately following this, the speed was gradually increased by 0.1 km/h every five cycles. Participants were instructed to indicate the point at which they felt unable to continue increasing the speed. They were encouraged to increase it and try to see whether they could maintain that pace. Participants were asked whether they could maintain it for 30 s; if not, they were allowed to decrease the speed. Additionally, if participants started moving away from the front handrail, the experimenter reduced the speed so that the measurement could begin. Once this threshold was reached, a final 30 s gait trial was conducted at the participant’s maximum safe walking speed. Since spatiotemporal and kinetic parameters are speed-dependent [22], to ensure the same experimental conditions between tests, the preferred speed was kept constant before and after adaptive training. A sampling rate of 100 Hz was used to acquire all data from the 94.8 × 40.6 cm pressure platform mounted in the treadmill. The validity and reliability of the Zebris system were previously confirmed [23,24]. In order to ensure safety, all participants wore a safety harness. Participants were strongly encouraged not to hold onto the handrail. Even though all enrolled participants walked without any aids on the ground, three of our subjects in the most fearful group did not want to walk without handrails. They did not feel stable and exhibited positional drift on the treadmill, failing to maintain a stable location. Therefore, they were allowed to touch but not to hold the handrail, and they were also asked to maintain an upright body position during walking to avoid influence on gait characteristics.

The dependent variables included: step length (the distance measured from the heel of the one foot to the heel of the other foot) normalized to body height (%); stride length (cm); step width (cm); stance phase duration (%); single limb support phase duration (%), which at the same time represented swing phase duration of opposite leg; double stance phase duration (%); cadence (step/min); walking speed (km/h); stride variability (coefficient of variation (%)—(standard deviation of the analyzed strides divided by the mean) × 100%)); maximum vertical force (calculated as the mean value across all steps) normalized to body weight (%BW) in three separate foot areas: the forefoot, midfoot, and rearfoot. All variables were recorded directly before and after the adaptive training.

### 2.2. Intervention

Motor adaptation is a process where movements need to be modified in response to changes in the task, environment, or organism itself [25]. In the current study, adaptive locomotion training was applied by means of a computer game (Zebris FDM-T Software, version 3.0.14) to examine whether practicing walking while performing various goal-directed leg movements, combined with redirecting attention away from conscious control of the movements, leads to changes in walking pattern in older adults with different fear levels. Participants viewed a real-time representation of their feet on a path within the game on a 50-inch screen positioned about 1.5 m in front of their eyes. The subjects were practicing obstacle avoidance, crossing, aiming with the foot at a predefined field within the path, and performing additional cognitive tasks (such as simple mathematical operations). These tasks required participants to adapt their step length, position of their foot during the swing and stance phase, and to walk at varying cadences. All participants played the game at the same difficulty level, meaning they followed an identical path and completed the same set of motor and cognitive tasks. Due to selecting the same game level, the ratio of time spent performing motor tasks to time spent simply walking along the path was the same for all participants. As preferred walking speed correlated with functional ability and balance confidence, as well as reflected both functional and physiological changes, in our opinion, this measure was the best for the normalization of gait training, particularly when psychological factors start to play a significant role. This approach allows for gait training to be tailored precisely to each participant’s functional capacity.

The training lasted 20 min; however, three of our most fearful participants could not proceed with the training for longer than 15 min due to perceived fatigue and discomfort. As they met the inclusion criteria, we decided to include their results in the analysis, as they represented their current maximal capabilities. 

A physiotherapist was always present during training and testing.

### 2.3. Data Analysis

Descriptive analyses of the participants’ characteristics were performed by calculating the means and standard deviations for the continuous variables. To examine associations between gait variables (dependent variable) and fear of falling (independent variable), since we assumed a monotonic but not necessarily a linear relationship between the variables and due to the ordinal nature of the FES variable, Spearman’s rank correlation was applied. Steiger’s method was applied to calculate the difference between two dependent correlations from different walking conditions [26]. To assess participants’ responsiveness to adaptive gait training stimulation within each fear level group, depending on the distribution characteristics of the variables, either a paired t-test or the Wilcoxon signed-rank test was employed. In this analysis, lateral parameters were analyzed globally, without separating the right and left sides. The level of significance was set at *p* < 0.05. All statistical analyses were performed using the Statistica software package, version 13.1 (TIBCO Software Inc., Palo Alto, CA, USA); differences between correlations were calculated using the calculator available online [26]. Effect sizes were calculated as either Cohen’s d or dz = Z/√N for Wilcoxon tests and were considered as 0.40–0.69 moderate, 0.70–0.89 strong, 0.90–1.0 very strong [27]. AI was used to verify the linguistic correctness of some sentences in the manuscript.

## 3. Results

### 3.1. Correlation of Fear of Falling with Gait Characteristics

**Figure 1:**
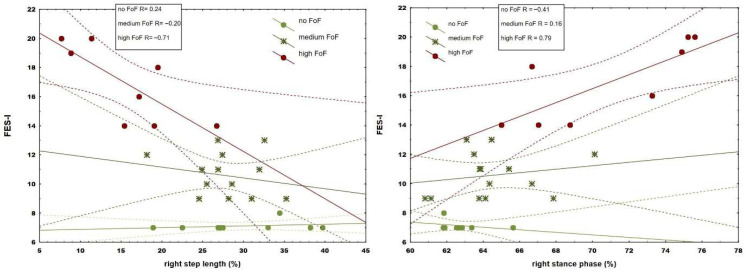
Regression plot of right step length (%) and right stance phase (%) in participants with varying levels of fear. Solid lines indicate the regression line, while dashed lines represent the 95% confidence interval.

**Figure 2:**
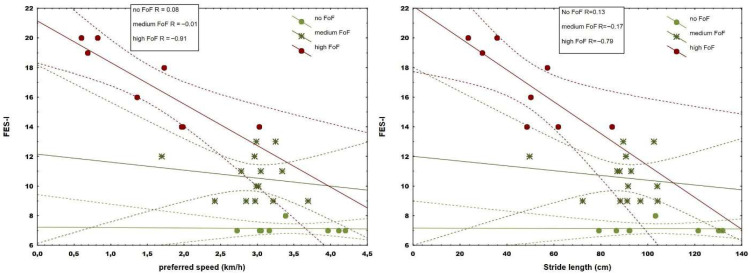
Regression plot of preferred walking speed (km/h) and stride length (cm) in participants with varying levels of fear. Solid lines indicate the regression line, while dashed lines represent the 95% confidence interval.

**Figure 3:**
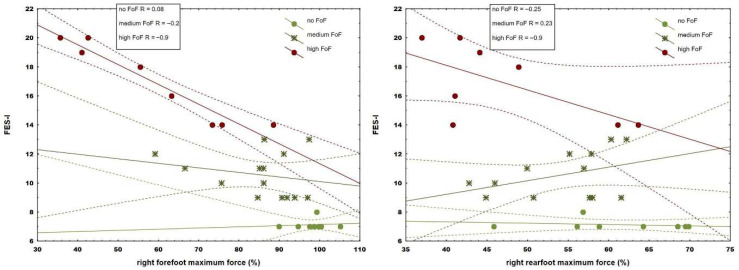
Regression plot of right forefoot vertical loading (%) and right rearfoot vertical loading (%) during walking with preferred speed in participants with varying levels of fear. Solid lines indicate the regression line, while dashed lines represent the 95% confidence interval.

Correlation analysis revealed moderate to strong associations with fear (range 0.66–0.74) in 9 from 11 spatiotemporal and 4 from 6 kinetic parameters (range 0.48–0.82) (Table 2) during walking with preferred speed, while 10 spatiotemporal (range 0.69–0.81) and 4 kinetic parameters showed moderate to strong associations (range 0.6–0.74) in more demanding walking conditions. Data from one participant (medium FoF) under maximum speed conditions were excluded from further analysis due to errors caused by technical issues. Although observed associations were more pronounced during walking with maximum tolerated speed, comparative analysis revealed that fear correlated significantly stronger in stride length (*p* = 0.03) and walking speed (*p* = 0.03), as well as forefoot (*p* = 0.01) and rearfoot loading (*p* = 0.02) when compared to preferred conditions (Table 2). When groups were separated into the three fear subgroups, only the most fearful group revealed strong correlations (from 0.63 to 0.91) between fear and spatiotemporal (step and stride length, stance phase duration, and gait velocity) (Figure 1 and Figure 2) and kinetic parameters in both walking conditions (Figure 3).

### 3.2. Adaptation After Gait Training Stimulation

#### 3.2.1. Gait with Preferred Speed

Analysis revealed significant improvement only in step width in the medium FoF group (t(13) = 5.13, *p* < 0.01, d = 1.37) during walking with preferred speed. The rest of the spatiotemporal gait parameters in each group remained unchanged (Table 3).

Forefoot loading significantly decreased after training in the no FoF group (t(15) = 2.91, *p* <0.01, d = 0.73), and force under the rearfoot significantly increased in the most fearful group (t(15) = −2.39, *p* = 0.05, d = −0.60) (Table 4).

#### 3.2.2. Gait with Maximum Speed

More significant changes were observed during walking in more challenging conditions (Table 3 and Table 4). In the no FoF group, stance phase decreased (t(15) = 2.60, *p* = 0.02, d = 0.65) and single leg support phase increased (t(15) = −2.64, *p* = 0.02, d = −0.66). The medium FoF group revealed the highest number of significant changes. Stride and step length increased (Z(12) = −4.18, *p* < 0.01, dz = 1.2; Z(26) = 4.10, *p* < 0.01, dz = 0.80 accordingly), stance phase decreased (t(26) = 2.43, *p* = 0.02, d = 0.48), and single leg support phase increased (t(26) = −2.40, *p* = 0.02, d = −0.47). Gait speed increased (t(12) = −3.24, *p* < 0.01, d = −0.90) and step width decreased significantly after training (t(12) = 2.73, *p* = 0.02, d = 0.76). The most fearful group also showed adaptive changes after training, including an increased step length (Z(15) = 1.96, *p* = 0.049, dz = 0.49), a shortened double support phase (t(7) = 2.93, *p* < 0.01, d = 1.03), and a shortened single support phase (Z(15) = 3.31, *p* < 0.01, dz = 0.83). The kinetic parameters did not change significantly in any of the groups.

## 4. Discussion

Our results show that fear of falling (FoF) was moderately to strongly associated with the deterioration of spatiotemporal gait parameters during walking at the preferred speed. This is consistent with results reported in previous studies [8,28,29]. In contrast to former results [29], in our study, the step width did not show a significant correlation with fear. However, as reported in that study, the mean step width in the most fearful group was narrower than in the no FoF group in the current study (9.05 ± 3.17 cm vs. 12.6 ± 3.9 cm, respectively). This discrepancy can stem from the fact that in previous studies, subjects walked overground, and in the current study, participants adopted a wider step in all groups, possibly due to treadmill conditions, which seem to be more difficult than walking overground. The mean step width in our medium FoF group was influenced by two subjects who, because of strong external rotation of the feet, were walking with a very narrow step width, which explains the reduced score for the medium FoF group. With respect to the kinetic parameters, fear was strongly related to decreased vertical forefoot loading, indicating decreased push-off phase dynamics. It was also moderately associated with decreased vertical force under the heel, suggesting reduced loading response phase dynamics. The observed decreased rearfoot loading cannot simply be explained by the fact that more fearful participants walked more slowly. An argument supporting this reasoning is the fact that, in the group of individuals with the highest level of anxiety, the structure of foot loading changed after a single gait adaptation training without changes in walking speed. These findings are in agreement with those of Brown et al. [30], who demonstrated that elderly individuals adopted an altered joint kinematic pattern as a result of the imposed postural threat and not due to changes in walking speed. Similar arguments arise from previous research [30], wherein authors suggested that older adults modified their gait strategy and control by increasing the distal muscle activation level (gastrocnemius m. and tibialis anterior m.) and by decreasing ankle angular motion and velocity when postural threat was increased. Similarly, Nagai et al. [31] reported an elevated ankle muscle co-activation during walking in participants with fear of falling when compared to the control group and described this behavior as a stiffening strategy, which seemed to enhance gait stability and could be a self-imposed adaptation as a consequence of increased fear of falling. Generally, loading under the midfoot did not correlate with fear; however, once the anxiety level grouping was considered, it became apparent that, in the high FoF group, greater anxiety levels were associated with increased midfoot loading. It suggests that, during walking at their preferred speed, participants with a high fear of falling increasingly loaded the whole foot during foot roll-over and/or the midfoot unloading, through the swing phase of the opposite leg, was less evident. This is consistent with the results previously mentioned in Brown et al. [30], where authors reported decreased joint angular velocities in the knee and hip in older adults under conditions with increased postural threat.

The correlation between fear and plantar loading during walking in older adults is a novel finding. These associations imply a more cautious foot roll-over pattern with increasing FoF. Furthermore, some gait changes became more apparent only at the highest fear level (Figure 1, Figure 2 and Figure 3), pointing to stronger, ongoing deterioration as FoF became more severe.

During walking in more challenging conditions, the relationship between fear and gait changes was more pronounced. These results suggest that the relationship between fear and detrimental gait changes scales to some extent with motor task difficulty. This could be attributed to several factors. From a stability and motor control perspective, walking on a treadmill at maximum speed was a more demanding task. Hence, fearful subjects could have engaged adaptive compensatory mechanisms (i.e., stiffening strategy) more than non- or less fearful older adults to mitigate postural deficits and reduce fall risk [31,32,33]. Secondly, it is well accepted that more demanding motor tasks require more attention and conscious control in older adults ([2,29]). Several researchers have suggested that under increased anxiety, individuals regress to earlier stages of skill development, where subjects reinvest cognitive resources and an inward focus of attention occurs. This results in the manipulation of conscious, declarative knowledge to control the movement execution of well-learned skills, leading to performance decrements [13,31,32,33]. Therefore, both mechanisms could have amplified detrimental gait changes in fearful participants in more demanding walking conditions. However, to directly clarify the contribution of these mechanisms, it would be noteworthy to examine older adults with different fear levels in challenging walking conditions, employing a dual-task paradigm and incorporating muscle activation analysis.

### Gait Adaptation

Learning new motor skills, as well as the modification of previously learned skills, is essential for everyday activities [17]. It is well accepted that motor sequence learning, along with motor adaptation, is preserved in older adults (for reviews, see [12,25]). However, it is also known that fear of falling exacerbates the detrimental effects of aging and potentially influences the learning capacity of fearful older adults [34]. Hence, the question of whether motor adaptation in participants with different fear levels remains, and the question of whether higher fear is associated with higher motor adaptation deficits remains unknown. The results showed that healthy older adults without fear of falling did not significantly change their spatiotemporal gait characteristics in preferred walking conditions. It suggests that participants adopted their optimal gait pattern before and stayed at their optimal level after 20 min practice, and/or the stimuli were subliminal. Only forefoot loading decreased significantly during walking at the preferred speed. From a biomechanical perspective, this outcome could stem from slightly different gait kinetic characteristics of treadmill walking in comparison to overground conditions. After 20 min of training, participants could adapt and decrease their push-off phase strength. This can be supported by previous studies, in which the authors reported that all peaks of ground reaction forces were found to be significantly smaller for treadmill gait when compared to normal overground gait [35].

During walking with maximum speed, only stance time and single limb support time improved significantly; however, these changes were less than 1% and fall within the 95% limits of agreement reported by Faude et al. [23], suggesting the effect may also reflect measurement error.

Among a group with average fear levels, the results indicate the highest number of spatiotemporal changes after adaptive training. In conditions with maximum speed, participants walked significantly faster. Improvements in step width, as well as step and stride length, fall clearly within the minimum detectable change reported previously, suggesting a meaningful change [23]. Also, most fearful subjects showed significant improvements during walking at maximum speed. A longer step length (mean difference of 4.3 cm), longer single limb support time, and shorter double support phase suggest that training imposed not only significant but also meaningful adaptation in their walking pattern [23]. These results showed that even short-term training leads to gait adaptation and significant changes in fearful older adults. Although the post-training values did not reach the level of the less fearful group, the effect sizes indicate that some spatial parameter gains were even greater than those observed in less fearful adults. These results lead to the conclusion that fear does not abolish the capacity for movement adaptation and learning in older adults. Future research could further explore the influence of fear on attentional processes involved in gait control, as well as the strategies (i.e., stiffening mechanism) adopted by fearful individuals under more challenging walking conditions. The question also arises as to whether longer or more intensive training would lead to greater adaptive changes in the most fearful older adults.

Several limitations in the current study must be addressed. One of them is a small sample size. Second, the study group was predominantly composed of female participants; hence, further research is needed to study differences between fearful men and women. Furthermore, the absence of a control group did not allow us to determine whether similar adaptive changes would have occurred without performing additional postural and cognitive tasks during treadmill walking. This represents a potential direction for future research. The final limitation is that we cannot answer the question of whether older adults without a fear of falling adopted an optimal gait pattern or if such a brief adaptive training was processed at a subliminal level. Hence, further studies incorporating varying durations of training stimulus exposure are required.

## 5. Conclusions

The correlation between fear and plantar loading during walking in older adults implies a more cautious foot roll-over pattern with increasing FoF. The results also suggest that the relationship between fear and detrimental gait changes scales, to some extent, with motor task difficulty. Importantly, the results also showed that fear does not abolish the capacity for gait adaptation in older adults, even following a very short-term adaptation process. However, this capacity is possibly attenuated in highly fearful older adults.

## Figures and Tables

**Table 1:** Group characteristic (Mean, standard deviation, and *p* values).

	No FoF	Medium FoF	High FoF	*p*
**age (yrs)**	67.4 ± 3.1	68.9 ± 3.7	70.5 ± 6.9	*p* > 0.05
**height (cm)**	168.8 ± 8.2	160.9 ± 7.5	163.5 ± 4.5	*p* > 0.05
**mass (kg)**	73.8 ± 9.8	75.1 ± 13.1	81.0 ± 19.9	*p* > 0.05
**sFES-I**	7.1 ± 0.33	10.6 ± 1.5	16.9 ± 2.7	***p* < 0.05**

sFES-I—short Falls Efficacy Scale International, FoF—fear of falling.

**Table 2:** Mean, standard deviation, and Spearman’s rank correlation (*Rw*) between fear of falling (FoF) and spatiotemporal as well as kinetic parameters under two different difficulty conditions.

		Preferred Walking Speed				Maximum Walking Speed				*p*
		No FoF	Medium FoF	High FoF	*Rw*	No FoF	Medium FoF	High FoF	*Rw*	
**step length (%)**	L	30.8 ± 5.6	27.6 ± 5.0	14.5 ± 5.7	***−0.66***	41.6 ± 4.6	38.8 ± 4.8	19.8 ± 9.3	***−0.72***	*p* = 0.12
	R	31.2 ± 6.0	27.8 ± 4.1	15.7 ± 6.3	***−0.69***	42.4 ± 4.9	38.8 ± 4.7	20.6 ± 9.3	***−0.74***	*p* = 0.17
*R (L vs. R)*		*0.10 0.24*	*−0.27 −0.20*	***−0.78 −0.71***		*0.00 −0.13*	*−0.41 −0.14*	***−0.77 −0.77***		
**stance phase (%)**	L	62.4 ± 1.6	64.2 ± 2.7	71.0 ± 5.8	***0.66***	60.5 ± 0.8	61.6 ± 2.1	68.4 ± 5.0	***0.71***	*p* = 0.15
	R	62.8 ± 1.3	64.5 ± 2.4	70.8 ± 4.4	***0.74***	60.1 ± 0.8	61.6 ± 1.9	67.9 ± 5.1	***0.76***	*p* = 0.34
*R (L vs. R)*		*−0.41 −0.41*	*0.21 0.16*	***0.63 0.79***		*0.0 −0.27*	*0.13 0.03*	***0.81 0.75***		
**single support (%)**	L	37.2 ± 1.3	35.5 ± 2.4	29.9 ± 4.1	***−0.73***	39.9 ± 0.8	38.5 ± 1.9	31.9 ± 5.2	***−0.77***	*p* = 0.24
	R	37.6 ± 1.6	35.8 ± 2.7	29.7 ± 4.7	***−0.67***	39.5 ± 0.8	38.4 ± 2.1	31.5 ± 5.1	***−0.71***	*p* = 0.16
*R (L vs. R)*		*0.41 0.41*	*−0.16 −0.26*	*−0.54 −0.5*		*0.27 0.0*	*−0.02 −0.14*	***−0.75 −0.80***		
**double st. (%)**		25.3 ± 2.5	28.7 ± 5.0	41.1 ± 8.9	***0.74***	20.7 ± 1.1	23.2 ± 3.9	36.4 ± 9.9	***0.75***	*p* = 0.33
*Spearman R*		*−0.58*	*0.21*	*0.52*		*−0.41*	*0.15*	***0.77***		
**step width (cm)**		12.6 ± 3.9	9.8 ± 2.6	12.1 ± 3.4	*0.06*	10.8 ± 2.5	8.7 ± 2.6	11.4 ± 3.9	*0.10*	*p* = 0.32
*Spearman R*		*0.00*	*0.39*	***0.77***		*−0.27*	*0.29*	***0.71***		
**stride len. (cm)**		104 ± 20.5	89.1 ± 14.0	48.9 ± 19.7	***−0.70***	145 ± 17.4	124.7 ± 15.0	65.9 ± 29.3	***−0.78***	***p* = 0.03**
*Spearman R*		*0.13*	*−0.17*	***−0.79***		*−0.27*	*−0.28*	***−0.77***		
**stride CV (%)**		2.8 ± 0.9	3.1 ± 1.3	10.4 ± 4.8	***0.67***	1.7 ± 0.7	2.1 ± 0.8	5.4 ± 2.8	***0.69***	*p* = 0.34
*Spearman R*		*0.41*	*0.21*	*0.15*		*0.54*	*0.11*	*0.43*		
**speed (km/h)**		3.5 ± 0.6	2.9 ± 0.5	1.5 ± 0.8	***−0.71***	5.5 ± 0.6	4.7 ± 0.7	2.3 ± 1.2	***−0.81***	***p* = 0.03**
*Spearman R*		*0.08*	*−0.01*	***−0.91***		*−0.14*	*−0.11*	***−0.86***		
		**Kinetic Parameters (vertical foot loading)**								
**forefoot (%)**	L	97.6 ± 3.2	84.7 ± 10.1	63.4 ± 15.8	***−0.74***	102 ± 10.9	89.4 ± 8.9	72.7 ± 19.8	***−0.67***	*p* = 0.13
	R	98.2 ± 4.4	85.1 ± 11.1	59.5 ± 19.0	***−0.82***	102 ± 12.3	90.7 ± 7.9	70.2 ± 23.3	***−0.60***	***p* = 0.01**
*R (L vs. R)*		*−0.08 0.08*	*−0.02 −0.2*	*−0.68 **−0.9***		*−0.54 −0.27*	*0.34 0.01*	*−0.64 **−0.80***		
**midfoot (%)**	L	25.9 ± 6.3	22.1 ± 5.5	32.2 ± 9.7	0.23	30.0 ± 8.5	26.2 ± 6.7	32.5 ± 8.6	*0.12*	*p* = 0.16
	R	25.1 ± 5.8	26.3 ± 6.7	31.7 ± 10.2	0.27	29.0 ± 8.7	29.0 ± 7.9	31.5 ± 8.4	*0.15*	*p* = 0.09
*R (L vs. R)*		*0.57 0.57*	*−0.4 −0.26*	***0.87 0.72***		*0.55 0.55*	*−0.24 −0.17*	*0.70 **0.72***		
**rearfoot** ***(%)***	*L*	60.3 ± 5.4	56.9 ± 10.0	43.0 ± 11.1	***−0.48***	85.1 ± 21.6	76.2 ± 12.0	49.9 ± 13.2	***−0.71***	***p* = 0.02**
	R	61.2 ± 8.4	52.2 ± 9.7	47.3 ± 9.9	***−0.49***	84.7 ± 16.9	73.3 ± 12.8	52.1 ± 11.5	***−0.74***	***p* = 0.02**
*R (L vs. R)*		*0.08 −0.25*	*0.24 0.23*	***−0.9*** *−0.45*		*−0.55 −0.55*	*−0.20 −0.1*	***−0.92*** *−0.52*		

L—left, R—right, *R (L vs. R)*—Spearman correlations for the left and right sides in each group, *p*—the significance level of the difference between the two correlations with FoF under different difficulty conditions (preferred vs. maximum speed), CV—coefficient of variation. Significant correlations are shown in bold.

**Table 3:** Mean, standard deviation, and *p* values for comparisons between before (pre) and after (post) adaptive training for spatiotemporal variables.

		Preferred Walking Speed			Maximum Walking Speed		
		No FoF	Medium FoF	High FoF	No FoF	Medium FoF	High FoF
**step length (%)**	*pre*	30.9 ± 5.6	27.7 ± 4.5	15.1 ± 5.8	42.8 ± 3.7	38.8 ± 4.6	20.2 ± 9.0
	*post*	31.1 ± 5.4	28.3 ± 4.1	15.8 ± 6.2	44.1 ± 4.4	41.0 ± 4.8	22.8 ± 11.4
		*p = 0.78*	*p = 0.11*	***p = 0.07***	*p = 0.11*	***p < 0.01 d_z_ = 0.80***	***p = 0.049*** ***d_z_ = 0.49***
**stance phase (%)**	*pre*	62.6 ± 1.4	64.4 ± 2.5	70.9 ± 4.9	60.3 ± 0.9	61.6 ± 2.0	68.2 ± 4.9
	*post*	62.5 ± 1.4	64.5 ± 2.5	70.5 ± 4.3	59.7 ± 0.9	61.3 ± 2.1	66.5 ± 5.0
		*p = 0.45*	*p = 0.16*	*p = 0.29*	***p = 0.02 d = 0.65***	***p = 0.02 d = 0.48***	***p < 0.01*** ***d_z_ = 0.82***
**single support (%)**	*pre*	37.4 ± 1.4	35.7 ± 2.5	29.8 ± 4.3	39.7 ± 0.9	38.4 ± 2.0	31.7 ± 5.0
	*post*	37.5 ± 1.4	35.5 ± 2.5	30.7 ± 3.1	40.3 ± 0.9	38.7 ± 2.1	33.5 ± 5.0
		*p = 0.47*	*p = 0.14*	*p = 0.06*	***p = 0.01*** ***d = −0.66***	***p = 0.02*** ***d = −0.47***	***p < 0.01*** ***d_z_ = 1.02***
**double stance (%)**	*pre*	25.3 ± 2.5	28.7 ± 5.0	41.1 ± 8.9	20.5 ± 1.1	23.2 ± 3.9	36.4 ± 9.9
	*post*	24.9 ± 2.7	29.0 ± 5.1	39.7 ± 6.5	19.4 ± 1.3	22.6 ± 4.1	33.0 ± 10
		*p = 0.5*	*p = 0.25*	*p = 0.28*	*p = 0.1*	*p = 0.09*	***p = 0.02 d = 1.02***
**step width (cm)**	*pre*	12.6 ± 3.9	9.8 ± 2.6	12.1 ± 3.4	10.8 ± 2.5	8.7 ± 2.6	11.4 ± 3.9
	*post*	11.9 ± 3.9	7.9 ± 2.3	11.9 ± 4.1	11.2 ± 2.9	7.8 ± 2.0	10.7 ± 4.5
		*p = 0.43*	***p < 0.01 d = 1.37***	*p = 0.82*	*p = 0.22*	***p = 0.02 d = 0.76***	*p = 0.44*
**stride length (cm)**	*pre*	104.5 ± 20.5	89.1 ± 14.0	48.9 ± 19.7	144.6 ± 17.4	124.7 ± 15.0	65.9 ± 29.3
	*post*	105.2 ± 20.7	91.0 ± 12.6	51.2 ± 21.1	149.0 ± 16.2	131.7 ± 16.2	74.6 ± 37.7
		*p = 0.8*	*p = 0.25*	*p = 0.17*	*p = 0.29*	***p < 0.01*** ***d_z_ = 1.2***	*p = 0.24*
**speed (km/h)**	*pre*	3.5 ± 0.6	2.9 ± 0.5	1.5 ± 0.8	5.5 ± 0.6	4.7 ± 0.7	2.3 ± 1.2
	*post*				5.6 ± 0.7	4.9 ± 0.8	2.7 ± 1.7
		-----	-----	-----	*p = 0.7*	***p < 0.01*** ***d = −0.90***	*p = 0.25*

FoF—fear of falling, *p*—significance level of the difference between pre- and post-adaptive training in each group during walking under different difficulty conditions, *d*—Cohen’s effect size, *d_z_*—effect size for the Wilcoxon test. Significant results are shown in bold.

**Table 4:** Mean, standard deviation, and *p* values for comparisons between before (pre) and after (post) adaptive training for the kinetic variables (vertical foot loading).

		Preferred Walking Speed			Maximum Walking Speed		
		No FoF	Medium FoF	High FoF	No FoF	Medium FoF	High FoF
**forefoot (%)**	*pre*	97.9 ± 3.7	84.9 ± 10.4	61.4 ± 17.0	101.5 ± 11.8	90.0 ± 8.3	71.5 ± 20.9
	*post*	95.7 ± 4.8	84.0 ± 11.2	58.3 ± 17.0	99.4 ± 9.8	90.7 ± 8.6	70.8 ± 21.5
		***p = 0.01 d = 0.73***	*p = 0.39*	***p = 0.07 d = 0.49***	*p = 0.21*	*p = 0.58*	*p = 0.61*
**midfoot (%)**	*pre*	25.5 ± 5.9	24.2 ± 6.4	31.9 ± 9.6	31.2 ± 7.3	27.6 ± 7.3	32.0 ± 8.2
	*post*	25.7 ± 6.0	25.3 ±6.4	32.4 ± 10.0	31.4 ± 8.9	28.2 ± 6.2	33.2 ± 10.2
		*p = 0.59*	*p = 0.15*	*p = 0.58*	*p = 0.80*	*p = 0.30*	*p = 0.13*
**rearfoot (%)**	*pre*	60.8 ± 6.8	54.5 ± 9.9	45.1 ± 10.4	84.4 ± 20.0	74.7 ± 12.2	50.9 ± 12.0
	*post*	60.7 ± 6.4	56.1 ± 8.0	48.5 ± 9.5	88.4 ± 9.1	76.5 ± 13.1	55.2 ± 16.8
		*p = 0.98*	*p = 0.16*	***p = 0.03*** ***d = −0.60***	*p = 0.44*	*p = 0.23*	*p = 0.13*

FoF—fear of falling, *p*—significance level of the difference between before (*pre*) and after (*post*) adaptive training in each group in two difficulty conditions (preferred vs. maximum), *d*—Cohen’s effect size. Significant results are shown in bold.

## Data Availability

The original contributions presented in this study are included in the Supplementary Material. Further inquiries can be directed to the corresponding author.

## Supplementary Materials

The following supporting information can be downloaded at: https://www.mdpi.com/article/10.3390/jcm14238311/s1, Table S1: spatio-temporal and feet loading data.

## Abbreviations

The following abbreviations are used in this manuscript:

FoF	Fear of falling
